# Determinants of surgeon choice in cases of suspected implant rupture following mastectomy or aesthetic breast surgery: Clinical implications

**DOI:** 10.1097/MD.0000000000021134

**Published:** 2020-07-02

**Authors:** Nicola Zingaretti, Emanuele Rampino Cordaro, Pier Camillo Parodi, Giulia Marega, Francesca Modolo, Carlo Moreschi, Ugo Da Broi

**Affiliations:** a Clinic of Plastic and Reconstructive Surgery; b Legal Medicine, Udine University Hospital, Department of Medical Area (DAME), University of Udine, Udine, Italy.

**Keywords:** breast implant rupture, breast reconstruction, informed consent, malpractice, medical liability

## Abstract

Implant ruptures may be diagnosed by physical examination, ultrasound (US), and magnetic resonance imaging (MRI). The absence of standard guidelines to approach to implant ruptures may cause unnecessary surgical revisions in the absence of radiological confirmation of prosthetic damages.

The purpose of this study was to analyze the diagnostic procedures applied to patients with suspected prosthetic rupture and surgeon choices to perform a revision or to plan a clinical and radiological follow-up.

We conducted a retrospective study on 62 women submitted to revision surgery due to radiological diagnosis of suspected implant rupture, following mastectomy or aesthetic reconstruction, and admitted to a Plastic Surgery Department between 2008 and 2018.

Seventy-three implants, believed to be ruptured, were explanted. One-third of these were intact and unnecessarily explanted. US associated with MRI evaluation resulted in the most helpful diagnostical method.

A standardized clinical and radiological approach is essential to manage breast implant ruptures successfully. An innovative protocol is proposed in order to: ensure the appropriate management of implant ruptures and prevent unnecessary surgical revisions; reduce the risk of claims for medical malpractice in cases of unsatisfactory final aesthetic results or worse than before.

## Abstract 2

Supplemental Digital Content is available in the text

## 1 Introduction

Capsular contracture and implant rupture are the most frequent postoperative complications of breast implantation.^[1]^ Iatrogenic damage, trauma, implant shell degradation, and mechanical pressure during mammography are among the reported mechanisms of implant rupture. Ruptures have been reported up to 15 years after implantation. ^[2–4]^

Breast implant ruptures are often silent and patients do not display any of the traditional symptoms such as changes in breast shape, size or firmness, capsular contracture, palpable lumps, or breast pain. However, if diagnosed, such complications demand careful clinical and radiological evaluation and a sound decision-making process in order to decide whether a prosthetic replacement is feasible or not.^[5]^

Correct treatment of implant rupture is also necessary to reduce the risk of claims for malpractice and compensation for damage suffered by the patient.^[6–10]^

The diagnosis of implant rupture is dependent upon imaging techniques such as mammography, ultrasound (US), and magnetic resonance imaging (MRI).^[11,12]^

In extracapsular rupture, both the implant shell and the fibrous capsule are breached, with macroscopic silicone leakage into the surrounding tissues or lymph nodes. Pathognomonic signs of extracapsular rupture are visible with both US and MRI.^[11]^

In intracapsular rupture, the implant shell is breached without macroscopic silicone leakage beyond the intact fibrous capsule. US reveals typical signs such as abnormal hyperechogenicity between the fibrous capsule and the prosthetic wall, which may show introflexion or stepladder signs while the signs revealed by MRI include the keyhole, noose, parallel line, pince-nez, “C” and linguine signs.^[13,14]^

As reported in the literature, there are fewer false positives in the diagnosis of extracapsular rupture than intracapsular rupture, whether US or MRI is used.^[13–15]^

A diagnosis of “suspected prosthetic rupture” is often possible by means of both US and MRI; in such cases, the radiologist will often employ only one of the 2 techniques and then advise the patient to consult a plastic surgeon.^[16]^

Furthermore, a diagnosis of suspected rupture in asymptomatic patients may cause the patient to experience anxiety and to complain of vague symptoms. This may in turn persuade the surgeon to perform revision surgery, even though the symptoms do not necessarily warrant such a course of action; in asymptomatic cases such as these, it is preferable for the surgeon, in agreement with the patient, to initiate a period of careful clinical monitoring while awaiting the prosthetic revision surgery.^[7–10,17–19]^

Owing to the lack of standard operative guidelines available in the literature, the aim of this work was to analyze the diagnostic and operative decisions made by surgeons in our University Hospital, in cases of confirmed or suspected prosthetic rupture in both symptomatic and asymptomatic patients, in order to identify the most critical aspects involving the management of such complications.

A practical protocol has been consequently designed, on the basis of the observational evidences, aiming to:

1. ensure the appropriate management of patients suffering from such complications
2. reduce the risk of demands for compensation from patients claiming for medical malpractice particularly in cases where the final aesthetic results are unsatisfactory or worse than before (owing to the intrinsic difficulties of such operations) and revision surgery reveals that there had in fact been no rupture of the implant.

## 2 Materials and methods

A retrospective observational and descriptive analysis was carried out between November 2008 and December 2018 on 62 patients who had undergone surgical removal of their prostheses following a diagnosis of suspected rupture. The women observed in our study had received their prostheses either following a mastectomy or after surgery performed for purely aesthetic reasons.

Patients who experienced both a suspected implant rupture and a recurrence of breast cancer were excluded from our study because in these cases radiological imaging cannot provide a clear, unambiguous diagnosis of prosthetic rupture.

Reported symptoms (pain, discomfort, breast deformation, redness) and main purposes of radiological investigations (follow-up/control, symptoms, trauma) were analyzed. The following data were screened: physical examination at presentation, radiological and surgical data.

Structural data of all removed prostheses involving material, texture, and Food and Drug Administration (FDA) guidances were analyzed.^[20]^

The study followed the principles of the Helsinki Declaration and was approved by the local ethics committee.

### 2.1 Evaluation of implant integrity

The results of US and MRI examinations requested by surgeon consultants were classified as follows:

1. *normal implant* when the shell showed no loss of continuity or when the typical signs of prosthetic rupture were absent
2. *suspected prosthetic rupture* when there were changes to the contour of the implant but no signs of rupture
3. *confirmed prosthetic rupture* when the shell showed a loss of continuity with the release of silicone from the implant.

The *confirmed prosthetic rupture* category was further subdivided into intracapsular rupture (where the silicone remained within the fibrous capsule), extracapsular rupture (where the silicone leaked outside the fibrous capsule into the surrounding breast tissue), and intraextracapsular rupture (when both features appeared).

### 2.2 Findings at explantation

After removal, the implants were examined for rupture, and classified as ruptured (intracapsular/extracapsular) or intact. Implants were considered to be normal when the elastomeric envelope was intact with no perforations, even when there was a thin layer of silicone caused by gel bleed; implants in which the elastomer shell presented a lack of continuity, peripheral fibrosis, or exudation were classified as ruptured. As requested by the manufacturer, the removed implants were sterilized and returned for further examination.

Any capsule irregularities were documented and a capsular biopsy was sent for pathological examination.

## 3 Results

We examinated 62 women (73 implants). Mean implant duration was 11.8 years (range 8–14 years).

All the removed prostheses were silicone gel made. The shape of removed implants was round (45% cases) and anatomical (55% cases) while their surface was both smooth (27% cases) and texturized (73% cases) (Table 1). The symptoms that the patients complained of, during the first follow-up with a plastic surgeon, were: pain and discomfort (28 cases: 45%), breast deformation (16 cases: 26%), and redness (4 cases: 7%); 14 the patients reported no pain (22%).

**Table 1:**
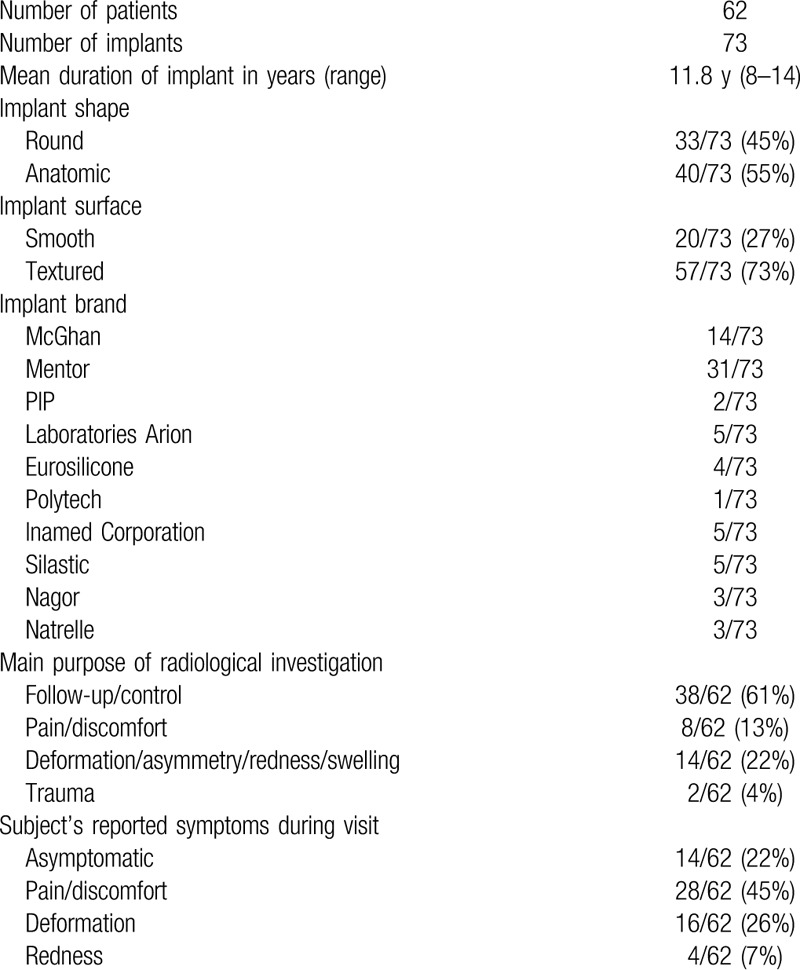
Implant characteristics of our cases suspected of rupture.

The clinical conditions necessitating radiological examination were: control/follow-up (38 cases: 61%), the presence of deformity-asymmetry and redness (14 cases: 22%), pain and discomfort (8 cases: 13%), and accidental trauma to the breast (2 cases: 4%).

The radiological procedures requested by surgeons were: ultrasound alone (40 cases: 55%), MRI alone (25 cases: 34%), MRI as a diagnostic follow-up after ultrasound diagnosis of suspected rupture (8 cases: 11%) (Table 2).

**Table 2:**
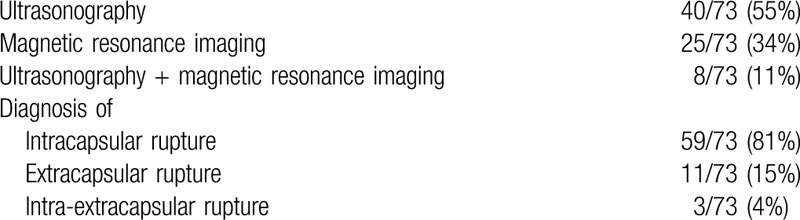
Evaluation of implant integrity.

The radiological investigations (using US, MRI, or a combination of both) showed: 59 cases intracapsular (81%), 11 cases extracapsular (15%), and 3 cases of intra and extracapsular rupture (3%).

At explantation 44 (60%) implants were found to be ruptured and 29 (40%) were intact (Table 3).

**Table 3:**
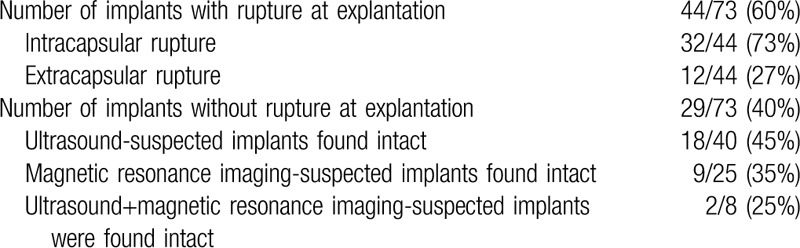
Findings at explantation.

Lastly, none of the capsules sent for pathological examination were positive for breast carcinoma or anaplastic large cell lymphoma.

## 4 Discussion

Breast implant rupture is one of the most serious complications and concerns for patients.

Both manufacturers and physicians commonly notify that breast implants are semipermanent. However, according to FDA classifications, breast implants have a limited product-life. A breast implant maintains its mechanical integrity for decades in a woman's body although the incidence of rupture increases with time.^[20]^

All the breast implants evaluated in our study had a CE (European Community) certification.

The majority of the removed prostheses (73%) were according to all FDA guidelines for material, texture, and labeling recommendations (i.e., Mentor, Natrelle, Inamed Corporation, McGhan prostheses).

Ruptures of prostheses may involve both smooth and texturized surfaces, both saline and silicone gel structures (the frequency of rupture is lower in case of silicone gel structure).^[21]^

In case of breast implants with a textured shell surface the producer employs a patented manufacturing process to make a specific textured surface so that all commercialized textured shells are structurally different.

It should be also taken into account that data from literature identified some patented prostheses having a frequency of rupture higher than other ones in use.^[22]^

The brand and “texturing process” of a prosthesis does not affect its radiological imaging and is not crucial for the clinical approach in cases of patients with a prosthetic suspected rupture.

Breast implant ruptures can be difficult to identify due to a frequent lack of symptoms and the different diagnostic and operative approaches employed by radiologists and surgeons.

Furthermore, there is no standardized approach to implant rupture, with the choice of radiological procedure (mammography, US, or MRI) and surgical procedure varying from one Breast Unit to another.

As reported by FDA guidelines, MRI is the most effective method for detecting silent rupture of silicone gel-filled breast implants.^[20]^ On the other hand MRI evaluation is expensive, so that MRI scans are generally planned for symptomatic patients or when suggestive findings are highlighted by US or mammogram.^[21]^

In Italian context asymptomatic suspected ruptures are diagnosed by US during normal follow-up checks and radiologists address them to MRI examination and surgical visit while symptomatic patients or suffering trauma undergo MRI directly.

Rietjens et al reported that MRI has an overall accuracy of 94%, compared with 72% for ultrasound and stated that MRI should be preferred when investigating possible implant rupture in women who had undergone mastectomy.^[7]^ Considering the higher costs of MRI, the authors admitted that US will continue to be used in many cases.^[5,11,16]^

As long as the patient is asymptomatic, yearly US examinations are sufficient, with MRI imaging every 5 years. However, in the case of symptomatic patients, MRI is preferable.^[5,12,16]^

US gives a higher percentage of false positives in asymptomatic patients and often results in unnecessary operations and increases the costs of screening and further surgery. MRI, on the other hand, is more accurate and sensitive in detecting rupture and should therefore be used for further screening if an abnormality is found.^[5,11,12]^

Our experience confirms that a selective use of MRI following US gives a lower percentage of false positives. The combination of both techniques can increase the sensitivity of radiological diagnosis and will result in fewer surgical operations and lower overall costs.

According to published data, our study confirmed that more than one-third of suspected implants were intact and unnecessarily explanted; when the prosthesis was found to be intact at explantation, a previous incorrect diagnosis of rupture resulted as already performed through US.^[4,14]^

Moreover, our experience confirms that the psychological discomfort of the patient may influence the surgeon's decision about the diagnostic and therapeutic procedure to be undertaken. It must be taken into account that near 50% of women report symptoms during follow-up or surgical visit after a radiological diagnosis: symptoms are often due to somatized psychological disturbances producing pain which may induce the surgeon to plan a prosthetic revision.

To date, there is no agreed standard diagnostic approach for identifying patients who require prosthetic revision surgery and clinicians, sometimes merely to avoid the risk of litigation, may carry out unnecessary prosthetic revisions.^[7–10,17–19]^ This can lead to complications, because the second operation can result in a poor aesthetic outcome.^[7–10,17–19]^ Such situations can lead to litigation and claims for compensation made by patients who are not satisfied with the aesthetic and/or functional results, especially when they learn that the implant was, in fact, intact and that the surgery was unnecessary.^[7–10,17]^

**Figure 1:**
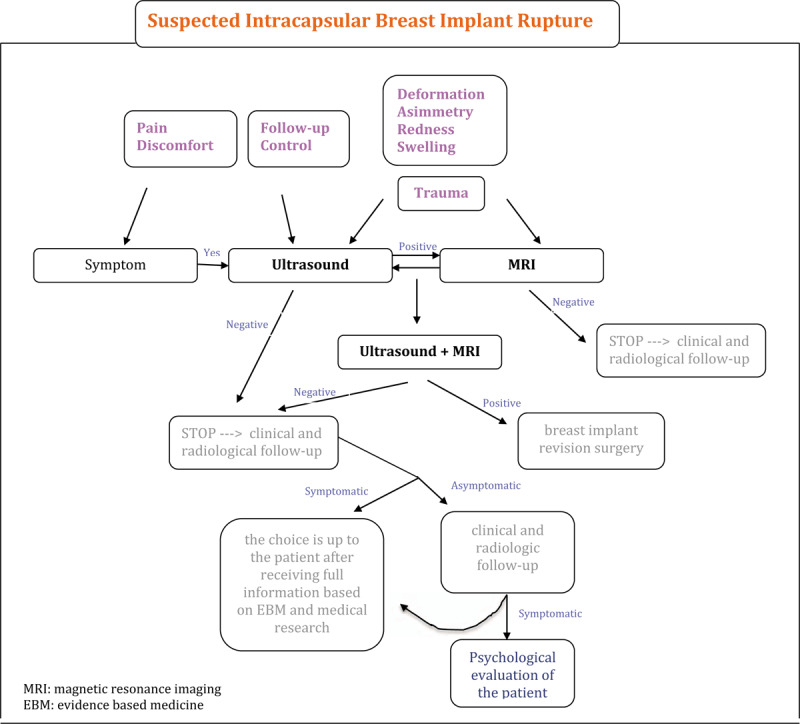
Flowchart. Asymptomatic patients request US at follow-up. Patients with symptoms or suffering trauma undergo MRI directly. Revision surgery is necessary when US and MRI confirm the prosthetic rupture. An interview with specific informed consent, based on EBM, allows patients to choose the best procedure in cases of unconfirmed prosthetic rupture as follows: asymptomatic subjects undergo radiological follow-up every 6 months (earlier if the clinical picture changes), symptomatic subjects receive psychological counselling and details about the pros and cons of surgery. EBM = evidence-based medicine, MRI = magnetic resonance imaging, US = ultrasound.

We have designed a practical protocol to ensure a most appropriate diagnosis of prosthetic rupture using a combination of US and MRI without unnecessary revisions: an innovative flowchart has been planned considering symptoms, US and MRI, psychological discomfort of the patient (see Fig. 1).

Extracapsular rupture is often associated with symptoms. In our study, rupture was confirmed during revision surgery in 91% of the cases where suspected extracapsular rupture was diagnosed.

In these cases US of the breast is the appropriate diagnostic procedure and there is no need to carry out MRI. From a medico-legal point of view, timely surgery is justified by the fact that a conservative approach or delayed surgery may expose the patient to an increasing risk of complications due to the rupture. Delayed surgery may mean that the operation becomes more complicated, with a far less certain outcome. Delays, therefore, could result in demands for increased damages: the disparity between the expected results of prompt surgery and those obtained from delayed surgery, involving aesthetic complications, may result in claims for malpractice. ^[7–10,17–19]^

Suspected intracapsular rupture diagnosed by US but not confirmed by MRI normally occurs in patients who undergo normal follow-up checks when the radiologist suspects, on the basis of ultrasound, that the prosthesis is ruptured. These patients normally are asymptomatic. From a clinical point of view, when the prosthesis is ruptured but the pathology is only progressing slowly, there is little expectation that the symptoms will worsen during the follow-up period. As Holmich et al suggested, implant rupture is a harmless event that does not seem to produce significant clinical symptoms or activate the humoral immune system. During the first 2 years following initial surgery there are very few cases of severe localized problems and/or rupture, which rather calls into question the belief that asymptomatic women should undergo explantation surgery.^[14]^ They proposed that women with asymptomatic implant rupture should have regular clinical check-ups, in order to assess any specific signs of free silicone migration, although there appears to be only a slight risk of this occurring.^[14]^ While it is essential to perform revision surgery when there is a definite diagnosis of implant rupture because of the high risk of complications, in cases of suspected rupture surgery can be delayed because of the low probability of complications during the follow-up phase. Moreover, from the surgical point of view, in case of intracapsular prosthetic no systemic or local risks rupture is possible if surgery is postponed.^[4]^ An early operation, on the other hand, especially on the basis of mere suspicion unsupported by clinical data, unnecessarily exposes a patient with an intact prosthesis to the risks inherent in surgery. It is preferable to give patients all necessary information regarding their clinical condition, and to explain that follow-up involving clinical-radiological monitoring means fewer potential complications. It is also important for patients with suspected ruptured implants to be informed that in case of complications may be attributable to the spread of free silicone (i.e., acute symptoms such as lumps, redness, soreness, or swelling), gradual silicone seepage may cause or exacerbate capsular contracture and the development of silicone granulomas. Such complications should be investigated by ultrasound scan and if this reveals a rupture, revision surgery will have to be carried out as a matter of the highest priority. ^[7–10,17–19]^

On the other hand, surgery is indicated if intracapsular rupture is diagnosed by US and confirmed by MRI because of the real risk that the clinical picture will deteriorate; however, since the risk of complications is low, especially when compared with cases of extracapsular rupture, the operation is not high priority.^[7–10,17–19]^ Unless there are complications or changes in the clinical picture, surgery can safely be performed within 90 days.

The emotional state of women diagnosed with a “suspected implant rupture” during a normal follow-up check (in most cases, therefore, asymptomatic) should not be underestimated as this can influence the diagnosis and, therefore, the subsequent therapeutic procedure.

It is mandatory to inform patients about the importance of a psychological counselling in such cases. If a patient whose symptoms are psychosomatic refuses psychological support, severe psychological discomfort may in itself be a sufficient indication for surgery, since it is the duty of the physician to protect both the physical and psychological health of the patient. Therefore, should the patient continue to insist on undergoing revision surgery, surgical intervention would be justified for the psycho-physical protection of the patient's health. From a medico-legal point of view, it is necessary in such cases to highlight the potential complications of reoperating, so that the patient is fully aware of the risks of a negative outcome following surgery, especially in those cases where the prosthesis is intact.

Before surgery it is essential to conduct a psychological interview, in order to ascertain the capacity of the patient to give consent and thereby avoid possible future disputes regarding the validity of consent granted by patient in a fragile psychological state.

In symptomatic patients with suspected intracapsular rupture diagnosed by US and MRI, detailed information must be given to the patient, including a description of the risks/benefits of the operation and the follow-up. If the patient continues to express a desire to undergo surgery, an intervention may be justified due to the presence of symptoms and because of the need to safeguard the woman's physical and psychological wellbeing. ^[7–10,17]^

A wide-ranging and exhaustive interview with the patient, to be done in all the above scenarios described, is of primary importance to be sure that her decision-making is founded on evidence-based medicine and medical research and that best practice will be applied: before giving her informed consent the patient must receive a guarantee that no unnecessary surgical treatment will be carried out.^[4,7–10,17–19]^

On the basis of our observational data we identified 5 possible scenarios and described related suggested procedures (see Supplementary Material – Annex 1).

### 4.1 Limitations of the Study

This study has some limitations to be pointed out:

1. clinical and, especially, radiological evaluations must always be regarded as highly physician-dependent.
2. radiological diagnosis was not evaluated by a second independent radiologist (as a second look procedure) before starting surgical removal of the prosthesis; this procedure is not mandatory in our Country.
3. Generally textured implants have a slightly higher failure rate than smooth-walled implants. The process of texturizing is somewhat traumatic and is thought to play a role in the increased leakage rate of the implant. However, the aim of our study was not to research possible correlations between the surface texturing and the prosthetic ruptures or to investigate the frequency of rupture in cases of different prosthetic brands; the aim of our study was to ensure the appropriate management of cases of suspected prosthetic rupture and reduce the risk of claims for medical malpractice.
4. All the removed prostheses were authorized and licensed by European Community laws, although some of them were not FDA licensed for material, texture, and labeling recommendations.^[23]^

## 5 Conclusion

Our experience, according to up-to-date clinical evidences, enabled us to highlight the inherent diagnostic and operative difficulties in cases of suspected breast implant rupture, due to the lack of standard published guidelines. We identified adequate investigative procedures to be applied to cases of breast implant rupture and a workable operative protocol which ensure the best outcome for patients and prevent the risk of legal claims for medical malpractice.

Further research is needed to confirm the utility of our proposed protocol for better management of suspected breast implant ruptures and reduce the risk of claims for compensation from patients for unsatisfactory aesthetic outcomes or for the unnecessary removal of a prosthesis.

A prospective study enrolling a larger number of patients will be necessary to validate this protocol and assess its long-term results.

## Author contributions

**Investigation:** Emanuele Rampino Cordaro, Pier Camillo Parodi, Giulia Marega, Francesca Modolo.

**Methodology:** Nicola Zingaretti.

**Supervision:** Carlo Moreschi, Ugo Da Broi.

## Supplementary Material

Supplemental Digital Content
