# Supplementary material for: Determinants of surgeon choice in cases of suspected implant rupture following mastectomy or aesthetic breast surgery: Clinical implications
Source: Medicine (Baltimore). 2020 Jul 2;99(27):e21134. doi: 10.1097/MD.0000000000021134 (PMC7337419; doi:10.1097/MD.0000000000021134)
Supplement: Supplemental Digital Content [file medi-99-e21134-s001.docx]

**Supplementary Material - Annex 1**

Possible scenarios and suggested procedures:

A: Extracapsular rupture diagnosed by US. This type of rupture is often associated with symptoms.

In such case:

- US of the breast is the appropriate diagnostic procedure
- There is no need to carry out MRI
- revision surgery should be performed as a matter of the highest priority.

B: Suspected intracapsular rupture (diagnosed by US but not confirmed by MRI) in an asymptomatic patient. In such case:

- MRI (which has a higher specificity) is necessary
- If the clinical picture is silent and yet rupture is still suspected even after MRI, immediate surgery is not necessary
- The patient is advised to wait and to repeat the ultrasound and MRI after six months
- Patients should be informed that acute symptoms such as lumps, redness, soreness, or swelling may be attributable to the spread of free silicone, and such complications should be investigated by US scan and if this reveals a rupture, revision surgery will be planned with highest priority.

C: Suspected intracapsular rupture (diagnosed by US and confirmed by MRI) in an asymptomatic patient. In such case:

- MRI (which has a higher specificity) is necessary
- If MRI confirms that the prosthesis is ruptured, surgery is recommended in order to avoid complications (not high priority; unless there are complications or changes in the clinical picture, surgery can safely be performed within 90 days).

D: Suspected intracapsular rupture (diagnosed by US) in an asymptomatic patient suffering from delayed symptoms. In such case:

- MRI (which has a higher specificity) is necessary
- If rupture is still suspected after MRI but the clinical picture is silent, a conservative approach is to be preferred, with no immediate need for surgery
- The patient should be advised to wait, remain vigilant and repeat the ultrasound and MRI after six months
- If, however, the patient complains of pain in the days following the examination, she should be:

a) told that pain alone does not necessarily indicate rupture

b) given detailed information regarding the potential risks/complications and advantages/benefits of both revision surgery and of a “wait and watch” approach

c) informed of the option of psychological counselling in order to identify a possible psychosomatic background to the disorders

- If a patient whose symptoms are psychosomatic refuses psychological support, severe psychological discomfort may in itself be a sufficient indication for surgery
- Detailed information must be given to the patient, including a description of the risks/benefits of the operation and the follow-up.

E: Suspected intracapsular rupture (diagnosed by US) in a symptomatic patient. In such case:

- MRI (which has a higher specificity) is necessary
- If the suspicion remains even after MRI, a close clinical-radiological monitoring should be recommended and only when the clinical-radiological picture becomes clear should revision surgery be performed
- Detailed information must be given to the patient, including a description of the risks/benefits of the operation and the follow-up
- Revision surgery should be performed if the patient continues to express a desire to undergo surgery.
